# Genetic analysis of *GABRB3* as a candidate gene of autism spectrum disorders

**DOI:** 10.1186/2040-2392-5-36

**Published:** 2014-06-25

**Authors:** Chia-Hsiang Chen, Chia-Chun Huang, Min-Chih Cheng, Yen-Nan Chiu, Wen-Che Tsai, Yu-Yu Wu, Shih-Kai Liu, Susan Shur-Fen Gau

**Affiliations:** 1 Department of Psychiatry, Chang Gung Memorial Hospital-Linkou, Taoyuan, Taiwan; 2 Department and Graduate Institute of Biomedical Sciences, Chang Gung University, Taoyuan, Taiwan; 3 Department of Psychiatry, National Taiwan University Hospital and College of Medicine, 7, Chung-Shan South Road, Taipei 10002, Taiwan; 4 Institute of Medical Sciences, Tzu Chi University, Hualien, Taiwan; 5 Department of Psychiatry, Yuli Mental Health Research Center, Yuli Veterans Hospital, Hualien, Taiwan; 6 Department of Child and Adolescent Psychiatry, Taoyaun Psychiatric Center, Ministry of Health and Welfare, Taoyuan, Taiwan; 7 Graduate Institute of Brain and Mind Sciences, and Graduate Institute of Epidemiology and Preventive Medicine, National Taiwan University, Taipei, Taiwan

**Keywords:** Autism spectrum disorders, *GABRB3*, Rare variants, Genetics, Case-control association

## Abstract

**Background:**

*GABRB3* is a position candidate gene at chromosome 15q12 that has been implicated in the neurobiology of autism spectrum disorders (ASD). The aim of this study was to examine the genetic association of *GABRB3* with ASD.

**Methods:**

The sample consisted of 356 patients with clinical diagnosis of ASD according to the DSM-IV diagnostic criteria and confirmed by the Autism Diagnostic Interview-Revised and 386 unrelated controls. We searched for mutations at all the exonic regions and 1.6 Kb of the 5′ region of *GABRB3* in the genomic DNA of all the participants using the Sanger sequencing. We implemented a case-control association analysis of variants detected in this sample, and conducted a reporter gene assay to assess the functional impact of variants at the 5′ regulatory region.

**Results:**

We detected six known common SNPs; however, they were not associated with ASD. Besides, a total of 22 rare variants (12 at 5′ regulatory, 4 at intronic, and 6 at exonic regions) were detected in 18 patients and 6 controls. The frequency of rare variants was significantly higher in the patient group than in the control group (18/356 versus 6/386, odds ratio = 3.37, *P* = 0.007). All the 12 rare variants at the 5′ regulatory region were only detected in 7 patients, but not in any of the controls (7/356 versus 0/386, Fisher’s exact test, *P* = 0.006). Two patients carried multiple rare variants. Family studies showed that most of these rare variants were transmitted from their parents. Reporter gene assays revealed that four rare variants at the 5′ regulatory region and 1 at exon 1a untranslated region had elevated reporter gene activities compared to two wild type alleles.

**Conclusions:**

Our data suggest rare variants of *GABRB3* might be associated with ASD, and increased *GABRB3* expression may contribute to the pathogenesis of ASD in some patients.

**Trial registration:**

Clinical trial registration Identifier: NCT00494754

## Background

Autism spectrum disorders (ASD) are a constellation of neurodevelopmental disorders characterized by the deficits in social reciprocity and language/communication ability, and the presence of restricted interests and repetitive behaviors [1]. The prevalence of ASD was estimated approximately as 1 per 110 children, with a male-to-female ratio of approximately 4:1 [2,3]. Genetic factors have been found to play an important role in the etiology of ASD [4-6].

Chromosome 15q11-q13 is a hot region of occurrence of genomic DNA deletions and duplications that are usually associated with developmental disorders including ASD [7-9]. For example, deletion of paternal segment 15q11.2-q12 is associated with Prader-Willi syndrome that is characterized by obesity, short stature, and hypotonia, while deletion of maternal segment of 15q11-13 is associated with Angelman syndrome which is characterized by mental retardation, movement disorder, and impaired language and speech development. Both Angelman and Prader-Willi syndromes are liable to have ASD [10]. Moreover, maternal duplication of 15q11-q13 was found in approximately 1 to 3% of patients with ASD [11]. Hence, genes located at this region have been considered to be potential candidate genes of ASD.

Gamma-aminobutyric acid (GABA) is the main inhibitory neurotransmitter in the brain. A cluster of GABA_A_ receptor subunit genes, including *GABRB3*, *GABRA5*, and *GABRG3*, which encode subunits β3, α5, and γ3, respectively, were mapped to chromosome 15q12 [8]. Several lines of study indicate that an altered GABAergic signaling pathway is associated with the pathogenesis of autism [12]. For example, reduced expression of GABA_A_ receptor subunits including *GABRB3*[13-17] and the GABA synthesizing enzymes, glutamic acid decarboxylase (GAD) 65 and 67 were found in several brain regions of patients with autism [18-20]. Mori and colleagues reported dramatically reduced GABA_A_ receptor binding in the superior and medial frontal cortex of patients with ASD using ^123^I-iomazenil (IMZ) single photon emission computed tomography [21]. These data render GABA_A_ receptor subunit genes potential candidate genes of autism.

Several genetic linkage and association studies have investigated the association of the three GABA_A_ receptor subunit genes at 15q11-13 with ASD. Cook and colleagues first reported linkage disequilibrium (LD) between autism and a genetic marker at *GABRB3*[22]. This finding was replicated by some studies [23-25], but not by others [26-29]. Menold and colleagues found two genetic markers in the *GABRG3* associated with autism [30]. McCauley and colleagues conducted a LD analysis of genetic markers spanning the 1-Mb of 15q12; they found six markers across *GABRB3* and *GABRA5* nominally associated with autism [31]. In view of the clinical heterogeneity of patients with ASD, several groups studied the genetic association of these GABA_A_ receptor subunit genes with subsets of ASD patients based on particular phenotypes. For examples, Shao and colleagues reported increased linkage of *GABRB3* locus with autism in families sharing the high insistence-on-sameness scores [32]. Similarly, Nurmi and colleagues reported improved linkage of *GABRB3* with autism in subset families based on savant skills [33]. Warrier and colleagues examined the association between 45 SNPs in *GABRB3* and Asperger syndrome; they found significant association of three SNPs with Asperger syndrome and multiple related endophenotypes of ASD [34]. Furthermore, Ma and colleagues investigated the genetic association of 14 known GABA receptor subunit genes and their interaction with autism. They concluded that the genetic interaction between *GABRA4* and *GABRB1* increased the risk of autism [35]. Investigating the interaction between the markers in four GABA_A_ receptor subunit genes in an Argentinean sample of ASD, Sesarini and colleagues found that the genetic interaction between *GABRB3* and *GABRD* was associated with an increased risk of autism [36]. However, Ashley-Koch and colleagues investigated the multi-locus effect of three GABA_A_ receptor subunit genes at 15q12 on autism but they did not find any positive association [37]. Atypical sensory sensitivity is one of the core features of patients with autism [38] and Tavassoli and colleagues found an association between genetic markers of *GABRB3* and tactile sensitivity in typically developing children, implicating the involvement of *GABRB3* in the atypical sensory sensitivity in autism spectrum conditions [39]. In addition, postmortem studies showed reduced *GABRB3* expression in patients with autism [15,16]. Taken together, converging evidence from these studies supports the idea that *GABRB3* may be an important candidate gene of ASD.

One study reported that mice deleted for all three subunit genes (Gabrg3, Gabra5, and Gabrb3) mostly died at birth with a cleft palate, and approximately 5% that survived exhibited neurological abnormalities. However, mice lacking the expression of Gabra5 or Gabrg3 did not show the neurological symptoms found in the mice lacking the three genes [40]. Furthermore, mice with deletion or reduction of Gabra5 showed enhanced learning and memory [41,42]. Mice lacking the *Gabrb3* had epilepsy phenotype and many behavioral abnormalities such as deficits in learning and memory, poor motor skills, hyperactivity, and a disturbed rest-activity cycle [43]. *Gabrb3* deficient mice also manifested a wide range of neurochemical, electrophysiological, and behavioral abnormalities that overlapped with the traits observed in ASD [44]. DeLorey and colleagues found that *Gabrb3* deficient mice exhibited impaired social and exploratory behaviors, deficits in non-selective attention and hypoplasia of cerebellar vermal lobules [45]. Hence, the phenotype of *Gabrb3* deficient mice was considered to represent a potential model of ASD [45]. Duplication of chromosome 15q11-q13 accounts for approximately 1 to 3% of autism cases [11]. A mouse model of 15q11-13 duplication showed several behavioral abnormalities that replicate various aspects of human autistic phenotypes [46]. However, the relevance of *Gabrb3* to the behavioral phenotypes has not yet been addressed in this animal model.

Prompted by these findings, we were interested to know whether *GABRB3* was associated with ASD in our population. The study specifically aimed to investigate whether there are variants at *GABRB3* that may confer increased risk to ASD in our population. To address this issue, we conducted deep sequencing of 1.6 Kb of the 5′ region and all exons and their flanking sequences of *GABRB3* in a sample of patients with ASD and control subjects from Taiwan using Sanger-sequencing. The *GABRB3* [GenBank: NG_012836.1] spans approximately 230 Kb at chromosome 15q12, and contains 10 exons. The first two exons (exon 1a and exon 1) are alternatively spliced exons of *GABRB3* that encode an open reading frame of 473 amino acids of isoform 2 and isoform 1, respectively. Although both isoforms have the same amino acid numbers, they have different amino acid sequences in their N-terminus. The sequencing approach basically followed the study conducted by Urak and colleagues with slight modification in the primer sequences [47].

## Methods

### Subjects

All the subjects enrolled into this study were Han Chinese from Taiwan. Patients with a clinical diagnosis of ASD made by board-certificated child psychiatrists according to the *Diagnostic and Statistical Manual of Mental Disorders-IV* (DSM-IV) [1] were recruited from the Children’s Mental Health Center, National Taiwan University Hospital (NTUH), Taipei, Taiwan; the Department of Psychiatry, Chang Gung Memorial Hospital (CGMH), Taoyuan, Taiwan; and Taoyuan Mental Hospital (TMH), Taoyuan, Taiwan. The clinical diagnosis of ASD was further confirmed by interviewing the caregivers (mainly parents) by qualified child psychiatrists using the Chinese version of the Autism Diagnostic Interview-Revised (ADI-R) [48,49]. The Chinese version of the ADI-R, translated into Mandarin by Gau and colleagues [48,49], was approved by Western Psychological Services in 2007. Their parents also reported their autistic symptoms on the Chinese version of the Social Responsiveness Scale (SRS), a 65-item rating scale with each item rated from 1 to 4 [50], and the Chinese version of the Social Communication Questionnaire (SCQ), a 40-item rating scale with each item rated as ‘yes’ or ‘no’ [49].

A total of 356 patients (312 boys and 44 girls, mean age: 8.84 ± 4.05 years) were recruited into this study. The ADI-R interviews revealed the 356 patients scored 20.65 ± 5.97 in the ‘qualitative abnormalities in reciprocal social interaction’ (cut-off = 10), 14.99 ± 4.21 in the ‘qualitative abnormalities in communication, verbal’ (cut-off = 8), 8.31 ± 3.25 in the ‘qualitative abnormalities in communication, nonverbal’ (cut-off = 7), and 14.99 ± 4.21 in the ‘restricted, repetitive and stereotyped patterns of behaviors’ (cut-off = 3). Ninety-five point one percent of the patients had abnormal development evident before 30 months of age. Among the 356 subjects with ASD, 17 have been diagnosed with epilepsy (4.78%), 5 have been suspected of seizure (1.40%), and 22 have had febrile convulsions (6.18%). Thirteen of them (3.65%) had been currently diagnosed with epilepsy.

The parents of patients with ASD were clinically assessed by well-trained interviewers who major in psychology and those whose children with ASD had genetic findings received psychiatric interviews by the corresponding author to confirm whether they had ASD. They also reported on the Chinese version of the Autism Spectrum Quotient (AQ) [51] to screen for any autistic trait and the Chinese version of Adult Self-Report Inventory-IV (ASRI-IV) [52] to screen for any psychiatric symptoms according to the DSM-IV diagnostic criteria.

A total of 386 unrelated control subjects (189 males, 197 females, mean age: 45.8 ± 12.5 years) were recruited from the Department of Family Medicine of Buddhist Tzu Chi General Hospital, Hualien, Taiwan. The current mental status and history of mental disorders of the control subjects were evaluated by a senior psychiatrist using the Mini-International Neuropsychiatric Interview [53]. Those with current or past history of mental disorders were excluded from the study. We did not check whether they had a family history of mental disorders.

The study protocol was approved by the Ethics Committee of each hospital (NTUH: 9561709027; CGMH: 93-6244; TMH: C20060905; ClinicalTrials.gov number, NCT00494754) and written informed consent was obtained from the participants and/or parents after full explanation of the protocol and reassurance of confidentiality and voluntary participation. Due to ethical consideration and human subject protection, we were not able to recruit gender- and age-matched control subjects into this study.

### PCR-based direct sequencing

Genomic DNA was prepared from the peripheral blood of each participant for PCR amplification. Thirteen amplicons that cover 1.6 Kb of the 5′ region and 10 exons of the *GABRB3* were generated from each individual and these amplicons were subjected to PCR-based autosequencing using ABI autosequencer 3730 (PerkinElmer Applied Biosystems, Foster City, CA, USA). Approximately 30 to 60 bp of the intronic region flanking the exon-intron junction of each exon were sequenced. All mutations identified in this study were confirmed by repeating PCR and sequencing. The primer sequences, optimal PCR conditions, and the size of amplicons are listed in the Additional file 1, and the locations of these amplicons are illustrated in the Additional file 2. The allele frequency of the variant greater than 1% was defined as common variation, whereas that less than 1% was defined as rare variation in this study.

### Reporter gene activity assay

Genomic DNA prepared from peripheral blood cells was used to construct the inserts for the reporter gene assay. For common and rare variants at the 1.6 Kb of the 5′ region, a sense primer containing the KpnI recognition sequence and an antisense primer containing the XhoI recognition sequence were used to PCR amplify the fragment from nucleotide positions −1,646 to −46 upstream to the ATG starting site of *GABRB*3 exon 1a (genomic DNA positions: chromosome 15: 27018917-27020517). The 1.6 Kb amplicon was first cloned into the pCR-Blunt II-TOPO vector (Invitrogen, Carlsbad, CA, USA), then subcloned into the pGL3-enhancer vector (Promega, Madison, WI, USA) using In-Fusion HD cloning kit (Clontech, Mountain View, CA, USA). For the mutation g.-53G > T at exon 1, a sense primer containing the KpnI recognition sequences and an antisense primer containing the XhoI recognition sequences were used for PCR amplification of a fragment of 597 bp from genomic DNA nucleotide positions: chromosome 15: 27017866-27018462. The amplicon was first cloned into the pCR-Blunt II-TOPO vector (Invitrogen, Carlsbad, CA, USA), then subcloned into the pGL3-enhancer vector (Promega, Madison, WI, USA). The authenticity of these clones was verified by Sanger sequencing.

Plasmids were transfected into an HEK293 or SKNSH cell line in 24-well plates using Lipofectamine 2000 (Invitrogen, Carlsbad, CA, USA) following the manufacturer’s protocol manufacturer. At 30 hours after transfection, cells were lysed and the luciferase activities were measured and normalized against *Renilla* luciferase using the Dual-Luciferase Reporter Assay System (Promega, Madison, WI, USA). Transfection of each plasmid construct was conducted in quadruplicate in each reporter gene experiment, and the reporter gene experiment was repeated three times. The ratio of firefly luciferase to *Renilla* luciferase was used to represent the normalized reporter gene activity of each construct. Comparison of reporter gene activity among different expression constructs was conducted using one-way analysis of variance (ANOVA) with *post-hoc* Tukey test. *P* < 0.05 was considered statistically significant.

### Bioinformatic analysis

Variants at the 1.6 Kb of the 5′ region were assessed by WWW Signal Scan (http://www-bimas.cit.nih.gov/molbio/signal/). Missense mutations were analyzed using PolyPhen-2 (http://genetics.bwh.harvard.edu/pph2/). Secondary structure analysis was conducted by CDM protein secondary structure prediction server (http://gor.bb.iastate.edu/cdm/). The evolutionary conservation of the mutation was evaluated using PMut (http://mmb.pcb.ub.es/PMut/). Synonymous mutations were assessed by RegRNA (http://regrna.mbc.nctu.edu.tw/index1.php) to predict their influence on splicing donor/acceptor sites. ESEFinder 3.0 (http://rulai.cshl.edu/cgi-bin/tools/ESE3/esefinder.cgi?process=home) was used to analyze exonic splicing enhancer motifs. The prediction of possible phosphorylation substrate motif and phosphorylation binding motif was analyzed using PhosphoMotif Finder (http://www.hprd.org/PhosphoMotif_finder).

### Statistical analysis

Deviation from the Hardy-Weinberg equilibrium was assessed by the Chi-square test. Genetic Power Calculator (http://pngu.mgh.harvard.edu/~purcell/gpc/) was used to perform a *post-hoc* power analysis. Linkage disequilibrium (LD) analysis was performed using Haploview version 4.2 [54] in which D’ was calculated using the method reported by Lewontin [55] and haplotype block was defined by the method described by Gabriel and colleagues [56]. Differences in allele, genotype, and estimated haplotype frequencies between patients and controls were evaluated using SHEsis (http://analysis.bio-x.cn/SHEsisMain.htm) [57]. Comparison of reporter gene activity among different expression constructs was conducted using one-way ANOVA with *post-hoc* Tukey test.

## Results

### Identification of common SNPs and association analysis

**Figure 1:**
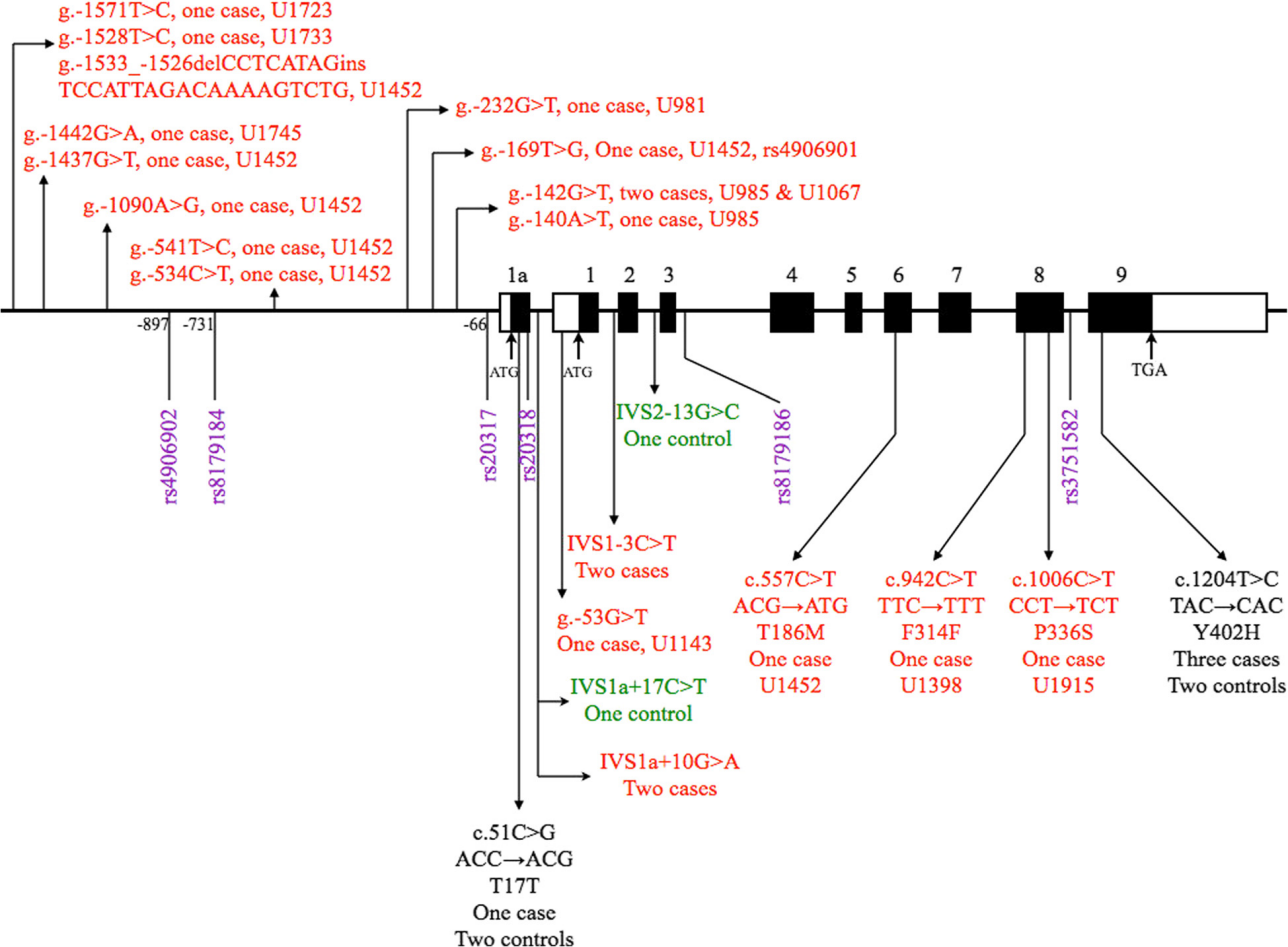
**Schematic genomic structure of the *GABRB3 *gene and the locations of genetic variants identified in this study.** The black boxes represent the protein-coding regions, while the open boxes represent the untranslated regions. SNPs identified in this study are listed below the schematic genomic structure and displayed in purple text. The positions of SNPs in the exon 1a promoter are numbered from the ATG start site. With regard to rare mutations, patient-specific mutations are displayed in red text, those discovered in controls only are displayed in green text, and those identified both in patients and controls are displayed in black text. IVS: intervening sequence; F: phenylalanine, T: threonine, S: serine, M: methionine, Y: tyrosine, H; histidine; del: deletion; ins: insertion.

We identified six known common SNPs in this study. The locations of these SNPs are illustrated in Figure 1. The detailed allelic and genotypic frequencies of these six SNPs, as well as the *post-hoc* power analysis are presented in Table 1. There were no significant differences in the frequency of allele or genotype of these six SNPs between patients and controls (Table 1). SNP rs3751582 was found to have a nominal association with ASD in genotype (*P* = 0.013, Table 1), but not in allele frequency analysis.

**Table 1:** Genotype and allele frequencies of the six known SNPs and association analysis between the patient (n = 356) and control (n = 386) groups

**Location**	**SNP ID**	**Group**	**Allele (1/2)**	**Genotype counts (frequency)**			***P *** **(df = 2)**	**Allele counts (frequency)**		***P *** **(df = 1)**	**Power (df = .1)**
				**1/1**	**1/2**	**2/2**		**1**	**2**		
5′ region	rs4906902	ASD	T/C	168 (0.472)	140 (0.393)	48 (0.135)	0.304	476 (0.669)	236 (0.332)	0.226	37.4%
		Control		161 (0.417)	171 (0.443)	54 (0.140)		493 (0.639)	279 (0.361)		
	rs8179184	ASD	G/A	168 (0.472)	141 (0.396)	47 (0.132)	0.342	477 (0.670)	235 (0.330)	0.244	37.3%
		Control		162 (0.420)	171 (0.443)	53 (0.137)		495 (0.641)	277 (0.359)		
	rs20317	ASD	C/G	167 (0.470)	140 (0.393)	49 (0.138)	0.327	474 (0.666)	238 (0.334)	0.347	37.6%
		Control		162 (0.420)	172 (0.446)	52 (0.135)		496 (0.643)	276 (0.357)		
Exon 1a	rs20318	ASD	C/T	167 (0.470)	139 (0.390)	50 (0.140)	0.220	473 (0.664)	239 (0.336)	0.406	37.7%
		Control		161 (0.417)	175 (0.453)	50 (0.130)		497 (0.644)	275 (0.356)		
Intron 3	rs8179186	ASD	C/T	168 (0.472)	141 (0.396)	47 (0.132)	0.367	477 (0.670)	235 (0.330)	0.289	37.4%
		Control		163 (0.422)	171 (0.443)	52 (0.135)		497 (0.644)	275 (0.356)		
Intron 8	rs3751582	ASD	G/A	195 (0.548)	126 (0.354)	35 (0.098)	0.013	516 (0.725)	196 (0.275)	0.131	34.0%
		Control		217 (0.562)	152 (0.394)	17 (0.044)		586 (0.759)	186 (0.241)		

Power indicates the estimates of power of risk alleles to detect the association with ASD under the assumptions of multiplicative inheritance mode, genotype relative risk (Aa) = 1.2, prevalence of disease = 0.01, D’ = 1 and *P*-value of 0.05 under null hypothesis. df indicates degree of freedom. ASD, autism spectrum disorders.

**Figure 2:**
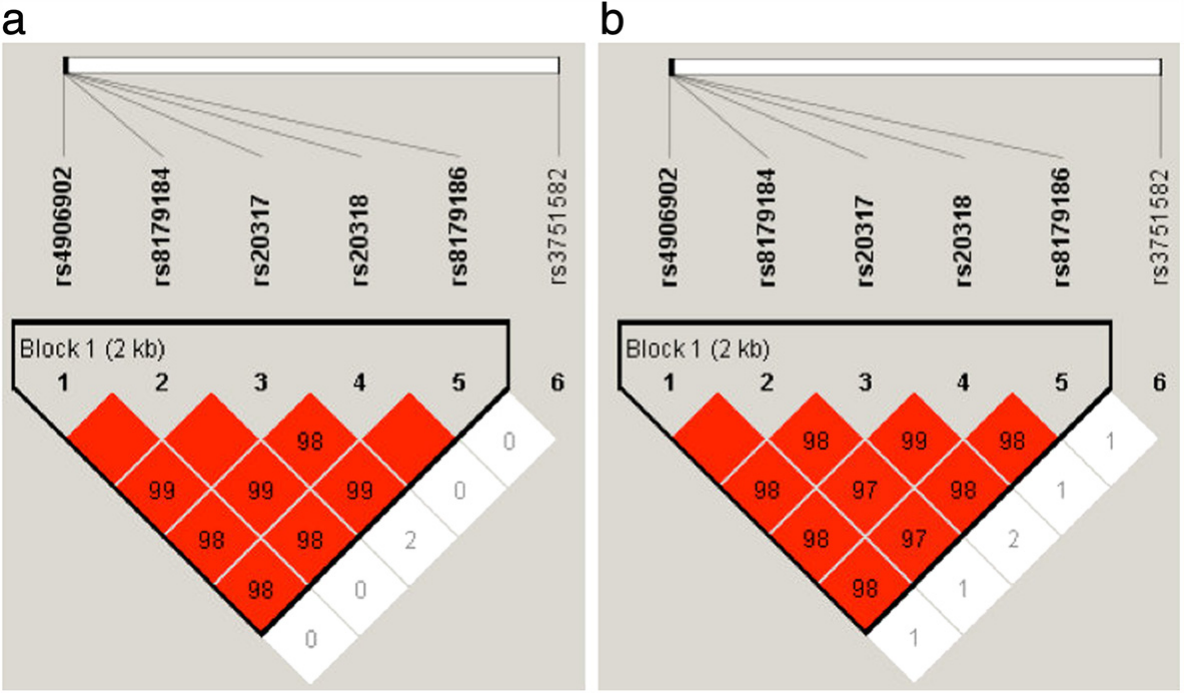
**Plot of pair-wise linkage disequilibrium (LD) analysis and haplotype block of six SNPs of *GABRB3 *identified in this study; (a) indicates LD plot of patient group, (b) indicates LD plot of control subjects.** D’ was shown in the plot.

Linkage disequilibrium (LD) analysis showed significant LD among five SNPs (rs4906902, rs8179184, rs20317, rs20318, and rs8179186) that formed a haplotype block in both patient and control groups (Figure 2). SNP rs20317 (g.-66C > G) was predicted to delete a myosin-specific/(+)GTCGCC transcription factor (TF) binding site (Additional file 3). Haplotype-based association analysis revealed four estimated haplotypes derived from these six SNPs, but they were not associated with ASD (Additional file 4).

### Detection of rare variants

Besides common SNPs, we detected a total of 22 rare variants in 18 patients and 6 control subjects, with a significant higher frequency in the patient group than the control group (18/356 versus 6/386, odds ratio = 3.37, *P* = 0.007) (Table 2). These 22 rare variants include 12 at the 5′ regulatory region, 4 at intronic regions, and 6 at exonic regions. Notably, all the 12 rare variants at the 5′ regulatory region were only detected in 7 patients, but not in any controls (7/356 versus 0/386, Fisher’s exact test *P* = 0.006) (Table 2). Locations of these rare variants and their distributions in patients and controls are illustrated in Figure 1.

**Table 2:** Comparison of the combined frequency of rare variants between the patient and control groups

**Rare variants**	**Patients (n = 356)**	**Control (n = 386)**	**Odds ratio**	***P***	
All rare variants	18	6	3.37	0.007	
5′ regulatory region	7	0		0.006	Fisher’s exact test
Intronic regions	4	2	2.18	0.36	
Exonic regions	8	4	2.20	0.19	

A patient had multiple mutations at the 5′ regulatory and a T186M missense mutation; hence, a total of 14 patients had the rare mutations.

### Family study of patients carrying the rare variants

**Figure 3:**
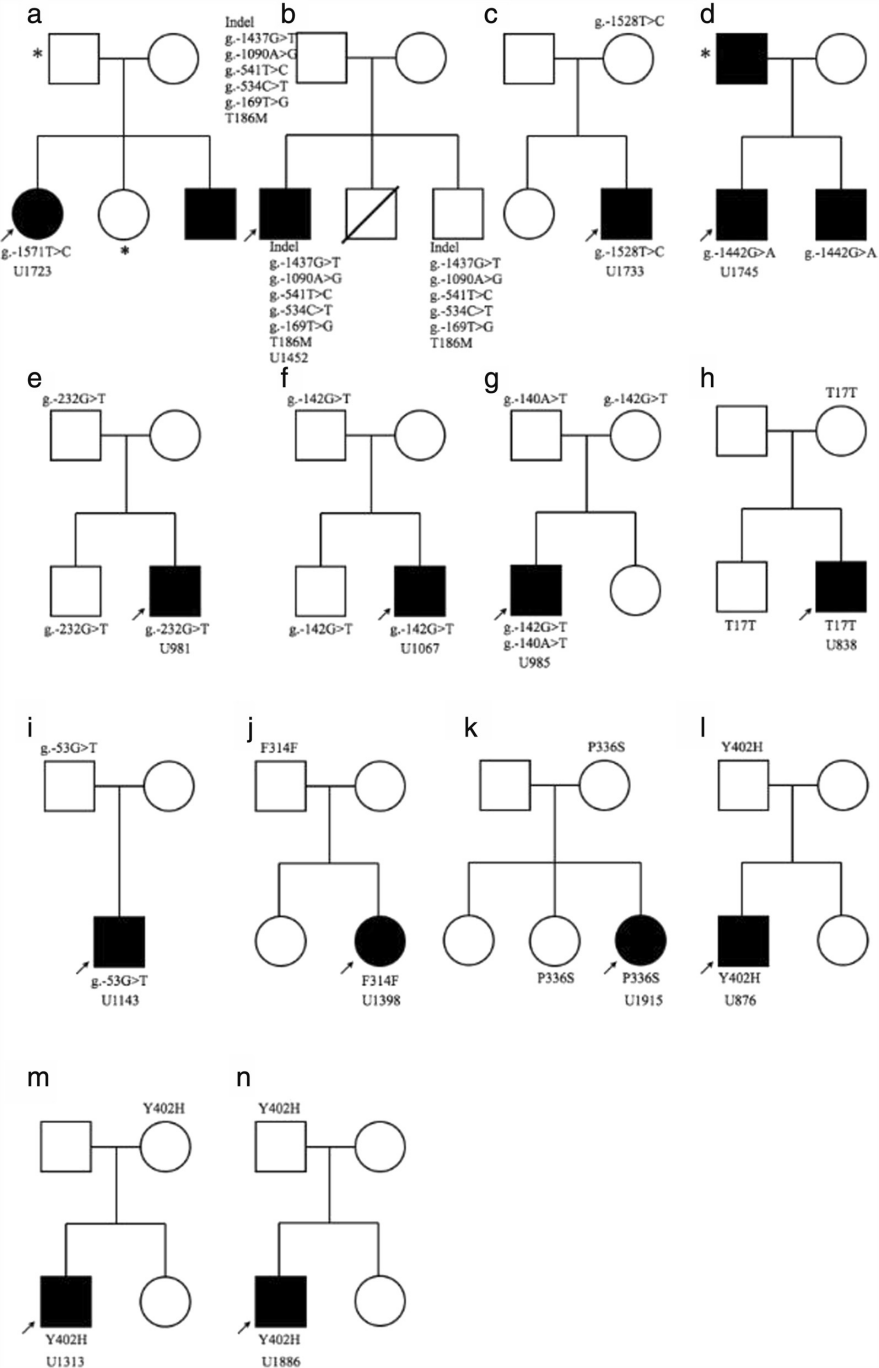
**Pedigrees for 14 autism spectrum disorder (ASD) families carrying rare variants at 5′ regulatory and the protein-coding regions of *GABRB3.*** Arrow indicates the proband and asterisk denotes individuals for whom DNA was not available. F: phenylalanine, T: threonine, S: serine, M: methionine, Y: tyrosine, H; histidine; del: deletion; ins: insertion.

A total of 14 families were enrolled for family study. Pedigrees of these 14 families are illustrated in Figure 3. The sex (11 males and 3 females), age (5.5 to 18.2 years old), full-scale IQ (57 to 140), subscores of the social reciprocity, verbal and non-verbal communication and behavioral domains assessed by the ADI-R, SCQ score, and SRS score of these 14 patients are summarized in Additional file 5. Based on the clinical assessment by the corresponding author, all the parents of the 14 patients did not have ASD except that the father of patient U1745 had ASD and the father of patient U1452 had autistic trait.

Starting from the 1.6 Kb of the 5′ end of the *GABRB3*, a g.-1571T > C variant was detected in a female patient (U1723, Figure 3a) with uncertain origin. This mutation was predicted to generate a new gamma-IRE_CS/(+)CTTGATCC TF binding site (Additional file 3).

Patient U1452 (Figure 3b) had a mutant allele harboring multiple variants at the 5′ regulatory region including an 11-bp insertion (g.-1533_-1526delCCTCATAGinsTCCATTAGACAAAAGTCTG), g.-1437G > T, g.-1090A > G, g.-541T > C, g.-534C > T, g.-169T > G and a T186M at exon 6. This complex mutant allele was transmitted from his father and found in another unaffected sibling. Based on clinical assessment, his father had some autistic trait characteristics such as abnormal reciprocal social interactions, difficulty finding common topics at conversation, lack of expression of his affection to or sharing with his family, and obsession with routine and rituals without meeting the DSM-IV diagnostic criteria. His report on the Chinese AQ (28) did not reach the cut-off (35) of very likely to have ASD in adults. Besides, he did not demonstrate any DSM-IV psychiatric symptoms except schizoid personality trait based on clinical assessment and his report on the Chinese ASRI-IV. All the variants at the 5′ regulatory region except g.-1437G > T were predicted to generate new TF binding sites (Additional file 3). The T186M missense variant at exon 6 was predicted to be ‘probably damaging’ (Additional file 3).

Patient U1733 (Figure 3c) had a g.-1528T > C variant that was transmitted from his unaffected mother. The variant was predicted to create a new TCF-1/(+) CACAG TF binding site (Additional file 3). Both patient U1745 and his bother suffered from ASD and had the g.-1442G > A variant (Figure 3d). This mutation was not detected in their mother. The father was clinically diagnosed as ASD by the corresponding author, a child psychiatrist. His key features since childhood included special interest in digits, objects, and patterns, stereotyped routine and interest, inflexible schedule, avoidance of any social interactions, difficulty figuring out what others wanted or were addressing, and socially inappropriate conversation or manners. His report on the Chinese AQ reached the cut-off of very likely to have ASD in adults (35 > cut-off as 32). He did not have any experience of imaginary play. He also demonstrated anxious, depressive, inattentive and schizoid symptoms based on clinical evaluation and his report on the ASRI-IV. However, the father refused blood withdrawal; therefore, his DNA was not available for testing. The g.-1442G > A was predicted to create three new TF binding sites (Additional file 3).

Patient U981 had a g.-232G > T mutation that was transmitted from his father and this variant was also found in his unaffected brother (Figure 3e). The g.-232G > T mutation was predicted to delete a HiNF-A/(+)AGAAATG TF binding site (Additional file 3). Patient U1067 had a g.-142G > T mutation that was transmitted from his unaffected father and this mutation was also found in his unaffected brother (Figure 3f). The g.-142G > T mutation was predicted to delete an AP-2/(+)CCGCCACGGC and create a new LBP-1/(+)TCTGG TF binding site simultaneously (Additional file 3). Patient U985 had two variants, g.-142G > T and g.-140A > T, that were transmitted from his unaffected mother and unaffected father, respectively (Figure 3g). The g.-140A > T was predicted to create a new LBP-1 TF binding site.

Patient U838 had a c.51C > G variant at exon 1a that was transmitted from his unaffected mother (Figure 3h). This variant was also detected in his unaffected brother and two controls. This variant did not alter amino acid sequence at codon 17 (T17T) and was predicted to have no functional influence (Additional file 3).

Patient U1143 had a g.-53G > T variant at the exon 1 untranslated region that was transmitted from his unaffected father (Figure 3i). The variant did not alter any TF binding site (Additional file 3).Patient U1398 had a c.942C > T variant at exon 8 that did not change phenylalanine at codon 314 (F314F) (Figure 3j). This variant was transmitted from her unaffected father and was not detected in any control subject in this study. This variant may influence the binding of the serine/arginine-rich splicing factor 6 (SRSF6) to an exonic splicing enhancer according to bioinformatic analysis.

Patient U1915 had a c.1006C > T mutation at exon 8 that changed the proline to serine at codon 336 (P336S) (Figure 3k). This mutation was transmitted from her unaffected mother, and was also found in her unaffected sister, but not in any control subject in this study. This variant may affect the secondary structure and the phosphorylation of the protein (Additional file 3).

A c.1204T > C variant resulting in amino acid substitution from tyrosine to histidine at codon 402 (Y402H) was found in three patients and two controls in this study. All three patients inherited this mutation from one of their parent dyads (Figure 3l-3n). This variant was already reported in the dbSNP with the number of rs185383468 (minor allele frequency = 0.005). Bioinformatic analysis predicted that this variant may affect the secondary structure and the phosphorylation of the protein (Additional file 3).

### Reporter gene assay

**Figure 4:**
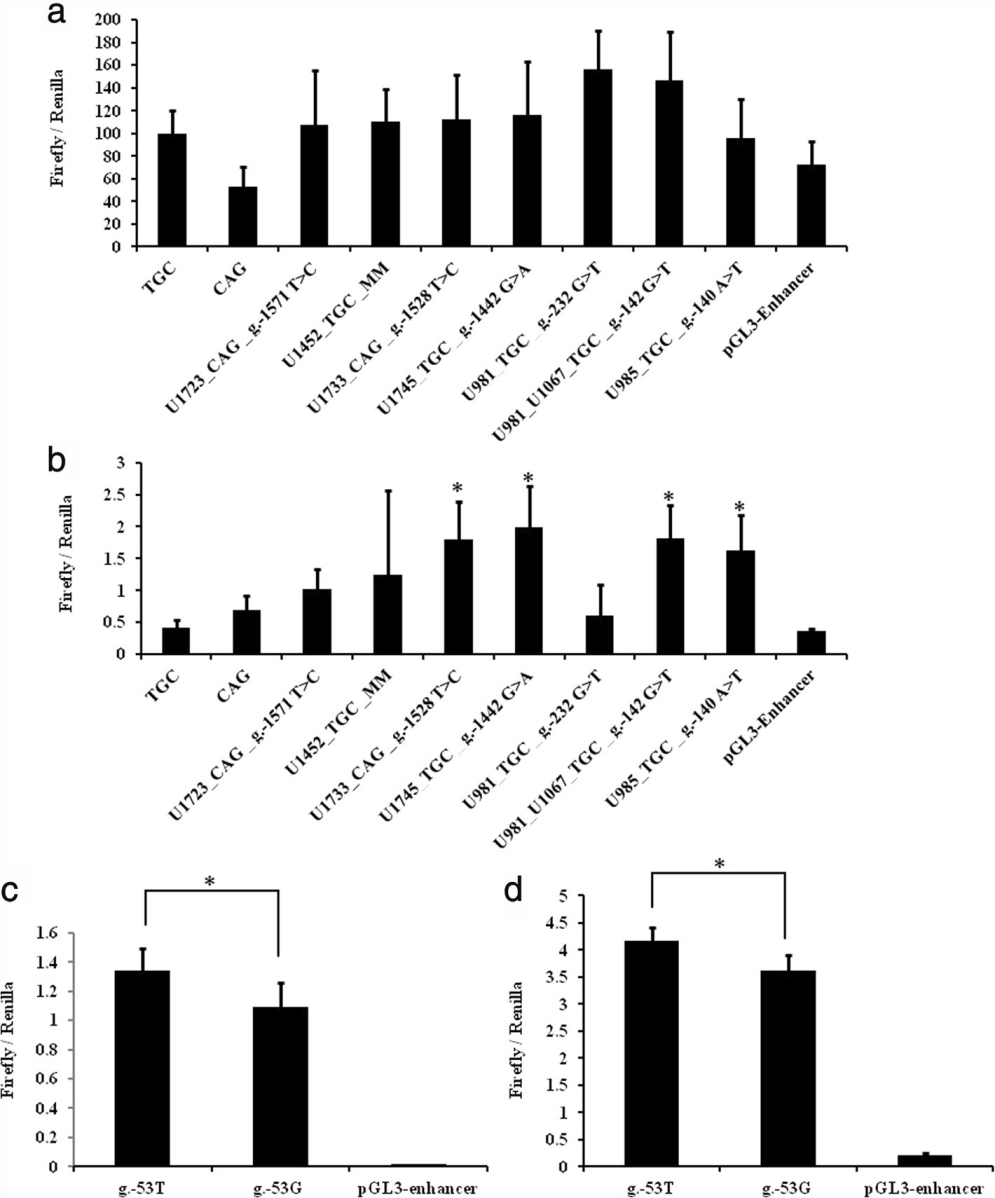
**Reporter gene activity assay of variants at the 5′ regulatory region and exon 1a untranslated region found in this study. (a)** The mean ratio and standard deviation of firefly luciferase activity to *Renilla* luciferase activity of each construct expressed in HEK293 cell line. No variant construct showed significant differences compared to both wild type constructs TGC and CAG simultaneously. **(b)** The mean ratio and standard deviation of firefly luciferase activity to *Renilla* luciferase activity of each construct expressed in SKNSH cell line. *indicates four mutation constructs including g-1528T > C (1.79 ± 0.60), g-1442G > A (1.99 ± 0.65), g.-142G > T (1.99 ± 0.65), and g-140A > T (1.62 ± 0.56) that showed significant elevation of reporter gene activity compared to two wild type constructs TGC (0.37 ± 0.66) and CAG (0.66 ± 0.50) simultaneously (*P* < 0.05). **(c)** The mean ratio and standard deviation of firefly luciferase activity to *Renilla* luciferase activity of the variant at exon 1a untranslated region measured in HEK293 cells. The mutant allele had significant elevated reporter gene activity (1.34 ± 0.16) compared to the wild type (1.09 ± 0.16; *P* < 0.05). **(d)** Similarly, the mutant allele showed significant reporter gene activity (4.18 ± 0.35) compared to the wild type (3.63 ± 0.28; *P* < 0.05) in SKNSH cells. MM indicates a multiple mutation that was found in one patient. TGC and CAG are two major haplotypes of the 5′ regulatory region found in this study.

The results of reporter gene activity assay are shown in Figure 4. In the experiment using the HEK293 cell line, the g-232G > T and g-142G > T showed significant elevation of reporter gene activity compared to the wild type construct CAG; however, they did not show significant differences in reporter gene activity when compared to the other wild type construct TGC (Figure 4a). In contrast, in the experiment using the SKNSH cell line, variants g-1528T > C, g-1442G > A, g.-142G > T, and g-140A > T showed significantly elevated reporter gene activity compared to both wild type constructs (TGC and CAG) (Figure 4b). In the assay of g-53G > T at exon 1 untranslated region, the mutant construct showed significant elevation of reporter gene activity compared to the wild type construct in both HEK293 (Figure 4c) and SKNSH cell lines (Figure 4d).

## Discussion

In this study, we identified a total of six common known SNPs in this sample; however, no association of these SNPs with ASD was detected. Due to the small sample size of this study, the study has only approximately 34 to 37% power to detect the association under the assumptions of multiplicative inheritance mode and the genotype relative risk of 1.2. But in view of the clinical heterogeneity of ASD, the possible association of these SNPs with some subsets of patients cannot be ruled out.

During the sequencing experiment, we detected only three common SNPs (rs4906902, rs8179184, rs20317) at the 1.6 Kb of the 5′ regulatory region in our sample. Urak and colleagues screened a sample of patients with childhood absence epilepsy for mutations in the 10 exons and the 5′ regulatory sequences of *GABRB3*. They found a total of 13 SNPs from the 5′ regulatory region to the beginning of intron 3. Four different haplotypes derived from these SNPs. Further, they found a SNP at the 5′ regulatory region which changed the binding of a neuron-specific transcriptional activator N-Oct-3 and showed different promoter activities in a reporter gene assay [47]. Several SNPs at this region, such as rs4273008, rs4243768, rs7171660, and rs4906901, as reported by Urak and colleagues in Austrians [47], were not detected in our sample. In addition, the rs4363842 as reported in Mexican and people in Honduras [58] was not found in the study of Urak and colleagues [47] and in our study. The three SNPs found in our sample also showed variations in frequency in different populations according to the dbSNP. Taken together, these findings suggest that there might be a population stratification of SNPs at the regulatory region of *GABRB3*, which should be taken into consideration when conducting a case-control association study of this gene. Among these three SNP, the rs20317 was shown to be located at the core promoter region of the *GABRB3* and the rs4906902 was located at the enhancer region. The C allele of rs20317 has been shown to have a significantly increased luciferase activity in a reporter assay [58].

A total of 22 rare variants were detected in this sample, including four variants (IVS1a + 10G > A, IVS1a + 17C > T, IVS1-3C > T, and IVS2-13G > C) located at intronic regions (Figure 1). The functional impact of these variants was predicted using bioinformatic analysis, and the results are listed in Additional file 3. The frequency of rare variants in the patient group was significantly higher than that in the control group, suggesting that rare mutations of *GABRB3* might be associated with ASD. Of note, all the rare variants at the 5′ regulatory region were detected in the patient group only in this study, with a significantly higher frequency in the patient group than that in the control group. This finding also implies that altered *GABRB3* gene expression might be involved in the neurobiology of ASD. The idea was partly supported by the reporter gene activity assay in this study. Four rare variants (g-1528T > C, g-1442G > A, g.-142G > T, and g-140A > T) at the 5′ regulatory region were shown to have elevated reporter gene activity compared to the wild type alleles. The results are compatible with our bioinformatic analysis that showed gain of transcriptional binding sites of these four variants except g.-142G > T. In addition to the gain of a LBP-1/(+)TCTGG, the g.-142G > T was also predicted to lead to a loss of an AP-2/(+)CCGCCACGGC binding site (Additional file 3). Our data are also compatible with the increased Gabrb3 expression seen in the chromosomal-engineered mouse model for human 15q11-13 duplication of autism [46]. *GABRB3* is biallelically expressed in control brain tissue samples. One study showed that the expression of *GABRB3* was subject to epigenetic alterations that resulted in monoallelic expression in a subset of autism [59]. A recent study reported significantly increased variance of *GABRB3* expression in the brain of patients with 15q11-13 duplication compared to control subjects and patients with autism [15]. Nevertheless, our findings are different from several postmortem studies that showed reduced *GABRB3* expression in the brains of patients with autism [14-16,60]. Hence, the clinical relevance of our findings in this study remains to be clarified.

In this study, we identified three missense mutations (T186M, P336S, and Y402H) and two synonymous mutations (T17T and F314F). The T17T is a new variant that was detected in one patient and two controls in this study, and appears to be neutral according to the bioinformatic analysis. The F314F was detected in only one patient but not in the controls in this study. Lachance-Touchette and colleagues reported the detection of the F314F synonymous variant in 1 out of 183 patients with idiopathic generalized epilepsy (IGE), but not in the 190 controls [61]. The authors suggested this variant might be pathological to IGE. This variant may influence the binding of the serine/arginine-rich splicing factor 6 (SRSF6) to an exonic splicing enhancer according to bioinformatic analysis conducted in this study, but the clinical relevance of this variant to ASD remains to be explored. The three missense mutations identified in this study, which occurred in the evolutionarily conserved region of *GABRB3*, may affect the secondary protein structure and alter a phosphorylation-based substrate motif or a phosphorylation-based binding motif. These findings suggest that these three missense mutations might have a functional impact on *GABRB3*.

Family studies revealed that almost all the rare variants found in patients were transmitted from their parents, and not all the carriers of rare variants met the clinical diagnosis of ASD, suggesting these rare variants are more likely to be risk factors rather than causative factors of ASD. Notably in the family of Figure 3g, the affected male patient (U985) carried two variants (g-142G > T and g.-140A > T) at the 5′ region of the *GABRB3* that were transmitted from his mother and father respectively, lending the evidence to support the multi-hit model of ASD [62,63].We also identified a novel mutant allele that harbored multiple mutations at the 5′ regulatory region and the T186M at exon 6 in the patient U1452 and his father (Figure 3b). Although the father did not meet the DSM-IV diagnostic criteria of ASD, he manifested autistic trait, suggesting this mutant allele might have a contributing effect to the pathogenesis of ASD. The T186M was predicted to be probably damaging in bioinformatic analysis. As the T186M was linked to the multiple variants at the 5′ regulatory region, and the reporter gene assay showed no significant differences in the reporter gene activity between the multiple regulatory variant alleles and the wild type constructs, the T186M alone might be the risk variant of ASD.

Missense mutations of *GABRB3* have been reported to be associated with childhood absence epilepsy (P11S, S15F, G32R) [64,65], insomnia (R192H) [66], and autism (P11S) [67]. In this study, we did not detect these missense mutations in our samples. In addition, among our patients with missense mutations, only the patient U1452 who carried the T186M mutation had a history of abnormal electroencephalography (EEG) in his childhood. The other patients did not have a history of seizure. These findings indicate that different mutations of *GABRB3* may confer different clinical presentations of neurodevelopmental disorders in their carriers [68].

## Conclusions

Our data suggest that rare variants of *GABRB3* might be associated with ASD, especially those at the 5′ regulatory region of *GABRB3*. Also, reporter gene activity assay of variants at the 5′ regulatory region suggests that increased *GABRB3* gene expression might be associated with the pathogenesis of ASD. Our study is limited by the small sample size and the lack of functional characterization of missense variants. Also, we found the ratio of firefly luciferase activity to *Renilla* luciferase activity to be small and not consistent across different experiments, suggesting that the expression construct may not work well in this study. Hence, our findings should be considered as preliminary and independent replication studies with a larger sample size are needed to verify our findings.

## Abbreviations

AABS_CS2: A-activator-binding site_consensus sequence 2; ADI-R: Autism Diagnostic Interview-Revised; ANOVA: analysis of variance; AP-2: activator protein 2; ASD: autism spectrum disorders; ASRI-IV: Adult Self-Report Inventory-IV; bp: base pair; CAP-site: catabolite activator protein site; DSM-IV: *Diagnostic and Statistical Manual of Mental Disorders, fourth edition*; EEG: electroencephalography; *GABRA5*: gamma-aminobutyric acid (GABA) A receptor, alpha 5; *GABRB3*: gamma-aminobutyric acid (GABA) A receptor, beta 3; *GABRG3*: gamma-aminobutyric acid (GABA) A receptor, gamma 3; GAD: glutamic acid decarboxylase; Gamma-IRE_CS: gamma-interferon response element_consensus sequence; GCF: GC binding factor; GMCSF_CS: granulocyte macrophage colony stimulating factor_consensus sequence; H4TF-1: histone H4 promoter transcription factor 1; H4TF-2: histone H4 promoter transcription factor 2; HiNF-A: histone nuclear factor A; IBP-1: insulin-like growth factor binding protein 1; IMZ: ^123^I-iomazenil; INF: interferon; LBP-1: leader-binding protein-1; LD: linkage disequilibrium; NF-kB: nuclear factor-kB; PCR: polymerase chain reaction; PR: progesterone receptor; PuF: c-myc purine-binding transcription factor; SCQ: Social Communication Questionnaire; SNP: single nucleotide polymorphism; SP1: specificity protein 1; SRS: Social Responsiveness Scale; SRSF6: serine/arginine-rich splicing factor 6; TCF-1: T-cell-specific transcription factor-1; TF: transcription factor.

## Competing interests

The authors declare no competing interests.

## Authors’ contributions

SSG is the principle investigator in this project. CHC and SSG designed the study and wrote the protocol. SSG trained the clinical research team, supervised in research execution, and collected all the clinical data of the ASD cases. SSG and YYW were responsible for the ADI-R training and interviews. SSG, YYW, YNC, WCT and SKL helped recruit and evaluate the patients and CHC screened for mental disorders in the controls. CCH and MCC conducted the experimental works and CHC supervised the experimental works and analyzed the data. CCH prepared the first draft, CHC and SSG critically revised the manuscript. All authors reviewed the article and approved its publication.

## Supplementary Material

Additional file 1 **Primer sequences, optimal annealing temperature (Ta) and size of amplicons of the *GABRB3*.** Click here for file

Additional file 2 **Locations of PCR amplicons in this study.** Double arrow head dashed line indicates the location of the amplicon. The amplicons for deep sequencing were shown in the upper panel of schematic genomic structure of *GABRB3*, while the amplicons for reporter gene assay were shown in the lower panel. E indicates exon. Click here for file

Additional file 3 **Summary of bioinformatic analysis of rare genetic variants of *GABRB3* identified in this study.** Click here for file

Additional file 4 Haplotype-based association analysis with ASD in this study. Click here for file

Additional file 5 **Demographic and clinical profiles of 14 patients with autism spectrum disorder who carried rare variants of *GABRB3*.** Click here for file

## Web resources

Primer3 software: http://biocore.unl.edu/primer 3. WWWSIGNALSCAN: http://www-bimas.cit.nih.gov/molbio/signal/. Polyphen-2: http://genetics.bwh.harvard.edu/pph2/. CDM protein secondary structure prediction server: http://gor.bb.iastate.edu/cdm/. PMut: http://mmb.pcb.ub.es/PMut/. RegRNA: http://regrna.mbc.nctu.edu.tw/index1.php. ESEFinder 3.0: http://rulai.cshl.edu/cgi-bin/tools/ESE3/esefinder.cgi?process=home. PhosphoMotif Finder: http://www.hprd.org/PhosphoMotif_finder. Genetic Power Calculator: http://pngu.mgh.harvard.edu/~purcell/gpc/.

Haploview: http://www.broadinstitute.org/scientific-community/science/programs/medical-and-population-genetics/haploview/haploview. SHEsis: http://analysis.bio-x.cn/SHEsisMain.htm
