# Supplementary material for: Genetic analysis of GABRB3 as a candidate gene of autism spectrum disorders
Source: Mol Autism. 2014 Jun 25;5:36. doi: 10.1186/2040-2392-5-36 (PMC4082499; doi:10.1186/2040-2392-5-36)
Supplement: Additional file 1 — Primer sequences, optimal annealing temperature (Ta) and size of amplicons of the GABRB3 . [file 2040-2392-5-36-S1.docx]

**Primer sequences, optimal annealing temperature (Ta) and size of amplicons of the *GABRB3* gene**

| Amplicon | Forward | Reverse | Ta (℃) | Size (bp) |
| --- | --- | --- | --- | --- |
| Exon 1a promoter1 | TCACAGGATCATCTTTGAGAGG | TGTGACCGCAGTACCTGAAA | 60 | 693 |
| Exon 1a promoter2 | CAGGGCATTTCTCCAAAAGA | TAGAAGGCTACTGGCGCACT | 60 | 689 |
| Exon 1a promoter3 | GAACACAAAAACGAGCTTGATG | GGTCCAGGAGAGCCAGATG | 60 | 769 |
| Exon 1a | GCTGGAAGACGGGTCAGG | CACGGGACTCGGACCTCT | 60 | 569 |
| Exon 1 promoter | CCATCTGGCTCTCCTGGAC | CTGGGAGAGGAAGGAGGAG | 60 | 574 |
| Exon 1 | GGTCCCAGAGGTCCGAGT | AACAGCTTGTCCACCGTCTC | 60 | 597 |
| Exon 2-3 | AAGGCTTTTCGGCATCTTCT | CACTGTGGACGCCTGTGAT | 60 | 613 |
| Exon 4 | CAACCTGAATTTGGCTGGTC | GAGGTCATTGCCTCACTTACAA | 60 | 400 |
| Exon 5 | CCACAGGGGCTTACTCGTT | TCTCTGTGTCTTCCCCTCTTTC | 60 | 300 |
| Exon 6 | AAACATAGCAGAGCCGCAAC | AGAGAGGAGGGGTGTGTGTG | 60 | 390 |
| Exon 7 | TCACCCCTTTATTCGCAGAC | AATGCTTGTGTCACGCTGTC | 60 | 385 |
| Exon 8 | TCCAGTCACCCCACAGTATTC | AAGGGTGTGTGGCTTGACAT | 60 | 495 |
| Exon 9 | AAAACGGAGTGCTGGATTTG | CTGCTTGTGTGTCTGTGTGTG | 60 | 585 |
