# Supplementary figures and images for: Genetic analysis of GABRB3 as a candidate gene of autism spectrum disorders
Source: Mol Autism. 2014 Jun 25;5:36. doi: 10.1186/2040-2392-5-36 (PMC4082499; doi:10.1186/2040-2392-5-36)

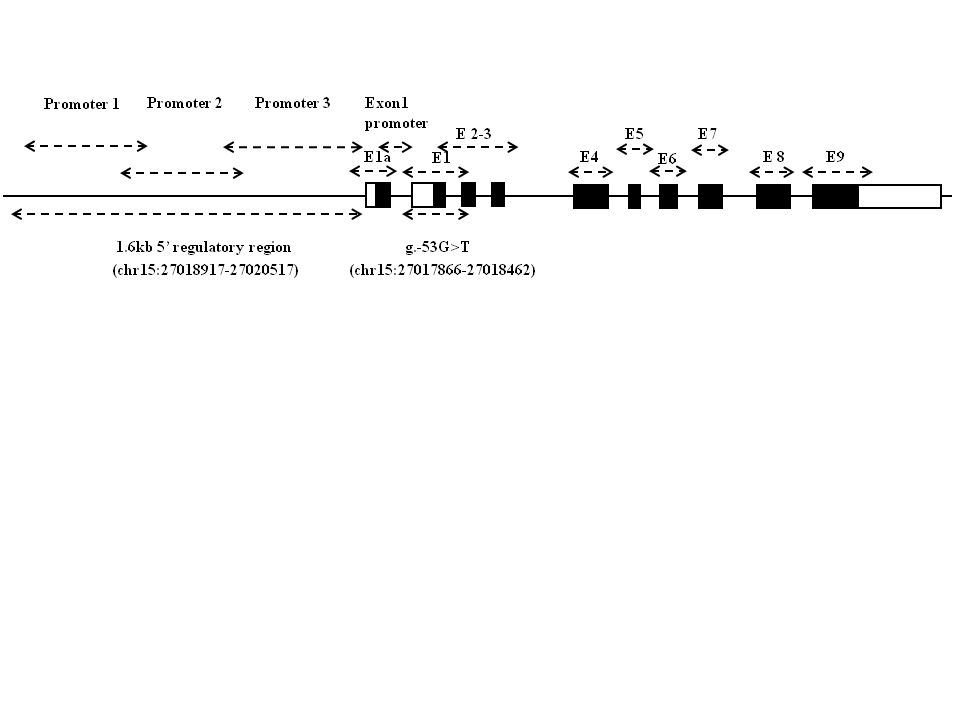

Supplement: Additional file 2 — Locations of PCR amplicons in this study. Double arrow head dashed line indicates the location of the amplicon. The amplicons for deep sequencing were shown in the upper panel of schematic genomic structure of GABRB3, while the amplicons for reporter gene assay were shown in the lower panel. E indicates exon. [file 2040-2392-5-36-S2.gif]
