# Supplementary material for: Genetic analysis of GABRB3 as a candidate gene of autism spectrum disorders
Source: Mol Autism. 2014 Jun 25;5:36. doi: 10.1186/2040-2392-5-36 (PMC4082499; doi:10.1186/2040-2392-5-36)
Supplement: Additional file 3 — Summary of bioinformatic analysis of rare genetic variants of GABRB3 identified in this study. [file 2040-2392-5-36-S3.docx]

**Summary of bioinformatic analysis of rare genetic variants of *GABRB3* identified in this study**

| Location | Nucleotide change | Amino acid change | *In silico* analysis | | |
| --- | --- | --- | --- | --- | --- |
|  |  |  | Transcription factor name/binding site/gain or loss | Polyphen-2  prediction | Change in secondary structure |
| 5’ region | g.-1571T>C | - | gamma-IRE_CS/(+)CTTGATCC/gain | - | - |
|  | g.-1533_-1526 delCCTCATAGinsTCCATTAGACAAAAGTCTG | - | gamma-IRE_CS/(+)CATTAGAC/gain; GMCSF_CS/(+)CATT/gain; TCF-1/(+)AAAAG/gain | - | - |
|  | g.-1528T>C | - | TCF-1/(+) CACAG/gain | - | - |
|  | g.-1442G>A | - | IBP-1/(+) AAGTGA/gain; INF.1/(+) AAGTGA/gain; alpha-INF.2/(+) AAGTGA/gain | - | - |
|  | g.-1437G>T  (rs4273008) | - |  | - | - |
|  | g.-1090A>G  (rs4243768) | - | AABS_CS2/(+)GTGATGCAA/gain | - | - |
|  | g.-897T>C (rs4906902) | - |  | - | - |
|  | g.-731G>A (rs8179184) | - |  | - | - |
|  | g.-541T>C  (rs7171660) | - | H4TF-2/(+)GGTCC/gain | - | - |
|  | g.-534C>T  (rs4363842) | - | SP1/(+)GGGCGG/loss; hsp70.2/(+)GGCGGG/loss; PuF/(+)GGGTGGG/gain | - | - |
|  | g.-232G>T | - | HiNF-A/(+)AGAAATG/loss | - | - |
|  | g.-169T>G (rs4906901) | - | CAP-site/(+)CAATTT/loss | - | - |
|  | g.-142G>T | - | AP-2/(+)CCGCCACGGC/loss; LBP-1/(+)TCTGG/gain | - | - |
|  | g.-140A>T | - | LBP-1/(+)TCTGG/gain | - | - |
|  | g.-66C>G (rs20317) | - | Myosin-specific/(+)GTCGCC/loss | - | - |
| Exon 1a | c.51C>G | T17T | - | - | - |
| Intron 1a | IVS1a+10G>A | - | GCF/(+)GCGGGGC/loss; NF-kB/(+)GGGGCTTCCC/loss; Sp1/(+)GGCGGG/loss | - | - |
| Intron 1a | IVS1a+17C>T | - | - | - | - |
| Intron 1a | g.-53G>T | - | - | - | - |
| Intron 1 | IVS1-3C>T | - | H4TF1/(-)GGGGGAGGG/loss | - | - |
| Intron 2 | IVS2-13G>C | - | PR/(+)TGTCCTCT/gain; TCF-1/(-)CTGTG/loss | - | - |
| Exon 6 | c.557C>T | T186M | - | Probably damaging | Yes |
| Exon 8 | c.942C>T | F314F | - | - | - |
| Exon 8 | c.1006C>T | P336S | - | Benign | Yes |
| Exon 9 | c.1204T>C | Y402H | - | Benign | Yes |
