# Supplementary material for: Genetic analysis of GABRB3 as a candidate gene of autism spectrum disorders
Source: Mol Autism. 2014 Jun 25;5:36. doi: 10.1186/2040-2392-5-36 (PMC4082499; doi:10.1186/2040-2392-5-36)
Supplement: Additional file 4 — Haplotype-based association analysis with ASD in this study. [file 2040-2392-5-36-S4.docx]

**Association analysis of haplotypes-derived from 6 SNPs identified in this study.**

| **Haplotype** | **Patient**  **(frequency)** | **Control**  **(frequency)** | **Monte Carlo p (Pearson)** |
| --- | --- | --- | --- |
| CAGTTA | 0.0898 | 0.0841 | 1.0000 |
| CAGTTG | 0.2360 | 0.2656 | 1.0000 |
| TGCCCA | 0.1827 | 0.1489 | 0.3860 |
| TGCCCG | 0.4775 | 0.4845 | 1.0000 |

Global Chi-square is 4.0140 while df=3 (frequency<0.03 in both control & case has been dropped); Permutation p value (Fisher) is 1.0000; Permutation p value(Pearson) is 1.0000.

**A**, Qualitative Abnormalities in Reciprocal Social Interaction, (cut off =10); **Bv**, Qualitative Abnormalities in Communication –Verbal (cut off =8); **Bn,** Qualitative Abnormalities in Communication –Nonverbal (cut off =7); **C**, Restricted, Repetitive, and Stereotyped Patterns of Behavior (cut off =3); **D,** Abnormality of Development Evident at or before 36 Months (cut off =1); **SRS**, the Chinese version of the Social Responsiveness Scale (65 items ; score 1-4 ; total scores, 65-260) ; **SCQ**, the Chinese version of the Social Communication Questionnaire (40 items; score 0,1 ; total scores, 0-40)
