# Supplementary material for: Genetic analysis of GABRB3 as a candidate gene of autism spectrum disorders
Source: Mol Autism. 2014 Jun 25;5:36. doi: 10.1186/2040-2392-5-36 (PMC4082499; doi:10.1186/2040-2392-5-36)
Supplement: Additional file 5 — Demographic and clinical profiles of 14 patients with autism spectrum disorder who carried rare variants of GABRB3 . [file 2040-2392-5-36-S5.docx]

**Demographics and clinical profiles of the 14 patients with autism spectrum disorder who carried rare variants of *GABRB3*.**

| **No.** | **ID** | **Sex** | **Age** | **Full-scale IQ** | Autism Diagnostic Interview-Revised | | | | | **SRS** | **SCQ** |
| --- | --- | --- | --- | --- | --- | --- | --- | --- | --- | --- | --- |
|  |  |  |  |  | **Past / Current** | | | | |  |  |
|  |  |  |  |  | **A** | **Bv** | **Bn^4^** | **C^5^** | **D^6^** |  |  |
| **a** | U1723 | F | 10.3 | 107 | 28/20 | 18/ - | 8/ - | 10/10 | 2 | 86 | 26 |
| **b** | U1452 | M | 11.6 | 105 | 27/ 9 | 12/ - | 8/ - | 7/2 | 4 | 104 | 16 |
| **c** | U1733 | M | 6.3 | 91 | 18/12 | 18/13 | 8/7 | 11/6 | 5 | 101 | 34 |
| **d** | U1745 | M | 10.0 | 140 | 10/7 | 8/ - | 2/ - | 7/7 | 4 | 84 | 23 |
| **e** | U981 | M | 18.2 | 113 | 25/8 | 21/ - | 14/ - | 6/2 | 5 | 69 | 22 |
| **f** | U1067 | M | 7.4 | 75 | 10/4 | 14/6 | 8/2 | 9/7 | 4 | 28 | 25 |
| **g** | U985 | M | 11.2 | 112 | 19/7 | 13/ - | 7/ - | 7/4 | 3 | 74 | 22 |
| **h** | U838 | M | 6.3 | 89 | 16/9 | 18/16 | 8/6 | 11/9 | 5 | 80 | 34 |
| **i** | U1143 | M | 7.6 | 111 | 26/14 | 19/15 | 11/8 | 12/12 | 4 | 105 | 24 |
| **j** | U1398 | F | 7.6 | 93 | 19/7 | 15/13 | 6/6 | 5/2 | 3 | 100 | 30 |
| **k** | U1915 | F | 7.9 | 57 | 26/23 | 21/18 | 13/10 | 9/9 | 5 | 91 | 19 |
| **l** | U876 | M | 11.3 | 126 | 20/6 | 16/ - | 8/ - | 7/3 | 3 | 87 | 14 |
| **m** | U1313 | M | 15.3 | 129 | 16/10 | 17/ - | 8/ - | 8/7 | 5 | 139 | 22 |
| **n** | U1886 | M | 5.5 | 114 | 7/5 | 6/5 | 2/2 | 2/1 | 3 | 79 | 28 |

**A**, Qualitative Abnormalities in Reciprocal Social Interaction, (cut off =10); **Bv**, Qualitative Abnormalities in Communication –Verbal (cut off =8); **Bn,** Qualitative Abnormalities in Communication –Nonverbal (cut off =7); **C**, Restricted, Repetitive, and Stereotyped Patterns of Behavior (cut off =3); **D,** Abnormality of Development Evident at or before 36 Months (cut off =1); **SRS**, the Chinese version of the Social Responsiveness Scale (65 items ; score 1-4 ; total scores, 65-260) ; **SCQ**, the Chinese version of the Social Communication Questionnaire (40 items; score 0,1 ; total scores, 0-40)
